# Progress in Methods for Copy Number Variation Profiling

**DOI:** 10.3390/ijms23042143

**Published:** 2022-02-15

**Authors:** Veronika Gordeeva, Elena Sharova, Georgij Arapidi

**Affiliations:** 1 Center for Precision Genome Editing and Genetic Technologies for Biomedicine, Federal Research and Clinical Center of Physical-Chemical Medicine of Federal Medical Biological Agency, 119435 Moscow, Russia; 2 Federal Research and Clinical Center of Physical-Chemical Medicine of Federal Medical Biological Agency, 119435 Moscow, Russia; sharova78@gmail.com (E.S.); arapidi@gmail.com (G.A.); 3 Moscow Institute of Physics and Technology, National Research University, Moscow Oblast, 141701 Moscow, Russia; 4 Shemyakin–Ovchinnikov Institute of Bioorganic Chemistry, Russian Academy of Sciences, 117997 Moscow, Russia

**Keywords:** copy number variation, karyotyping, chromosome microarray analysis, long-read and short-read sequencing

## Abstract

Copy number variations (CNVs) are the predominant class of structural genomic variations involved in the processes of evolutionary adaptation, genomic disorders, and disease progression. Compared with single-nucleotide variants, there have been challenges associated with the detection of CNVs owing to their diverse sizes. However, the field has seen significant progress in the past 20–30 years. This has been made possible due to the rapid development of molecular diagnostic methods which ensure a more detailed view of the genome structure, further complemented by recent advances in computational methods. Here, we review the major approaches that have been used to routinely detect CNVs, ranging from cytogenetics to the latest sequencing technologies, and then cover their specific features.

## 1. Introduction

Ever since Joe Hin Tjio and Albert Levan first identified the exact number of chromosomes in human cells in 1956, there have been massive advances in the understanding of the human genome and its structure [1]. In earlier times, due to the low resolution of methods for genetic material analysis, any form of chromosomal rearrangement was associated with disease or an abnormal condition. With advancements in the field after the completion of the human genome project, the use of comparative genomic hybridization (CGH) arrays has led to the discovery of an abundance of copy number variations (CNV), a structural variation of the DNA sequence which has a >50 bp multiplication and deletions of a particular segment of DNA, in the human genome [2,3]. It was shown that CNVs are widespread in human populations and comprise about 5–10% of the genome [4]. There is growing evidence showing the role of CNVs in causing various disorders. The list of diseases for which association with copy number variations has been established includes schizophrenia, type I diabetes, autism, cardiovascular diseases, congenital abnormalities, and neurodegenerative diseases [5]. However, due to the wide range of possible lengths and non-trivial estimation of the effect of CNVs on phenotype, there are difficulties in accurately screening and detecting CNVs. Due to limited resolution, modern technologies do not allow for complete CNV profile descriptions. However, each new approach to CNV detection has introduced new, valuable improvements. For example, chromosomal microarray analysis has allowed for large-scale genome studies with high resolution; whole-genome sequencing has provided the tool for the identification of all types of structural variations starting from 50 bp; and emerging sequencing technologies have opened up possibilities for the investigation of regions that were poorly accessible due to short reads. An enormous amount of copy number analysis methods using various types of data have been elaborated, and most of them have been described in detail in early reviews dedicated to a particular single technology or several CNV detection technologies [6,7,8,9]. Recently, the ELIXIR human CNV Community was initiated and focuses primarily on detection, annotation, and variant interpretation issues, which will ultimately help in developing an optimal protocol for CNV identification [10].

Hence, the aim of this review is to highlight the major contributions made in the field to detect CNVs to date and describe their main principles and challenges. We will also describe the most widely used computational approaches and discuss burning issues in this research field.

## 2. Methods of Cytogenetics

The first approaches to CNV detection were based on the analysis of metaphase plates in cells. During metaphase in cell division, condensed chromosomes align along the cell equator, facilitating convenient viewing of chromosome structure through light microscopy. Until the early 1970s, the only method for chromosome staining was Giemsa staining. In such an approach, all chromosomes are stained evenly length-wise, allowing for changes in karyotype concerning chromosome number, shape, and size to be visualized. Giemsa staining was used to detect many chromosome aneuploidies, such as Down’s syndrome [11], Klinefelter’s syndrome [12], and Edwards syndrome [13], as well as the structural abnormalities of chromosomes in cancer cells [14].

**Figure 1:**
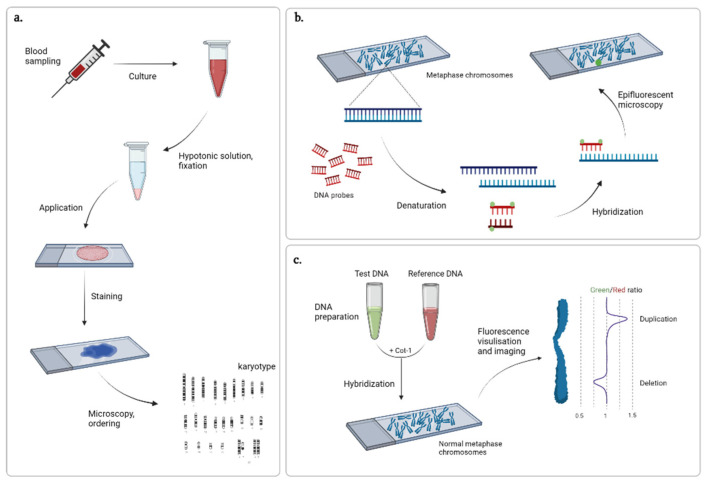
Cytogenetic techniques: (**a**) karyotyping, (**b**) FISH, and (**c**) comparative genome hybridization. Created with BioRender.com (accessed on 9 February 2022).

After the advent of Giemsa staining, new methods of differential staining that allowed for visualization of certain chromosomal structures, e.g., C-banding for centromere or T-banding for telomere regions staining, as well as producing chromosome-specific sequences of dark and light bands along the length (R, Q, and G-banding) were developed. The most common was the G-banding technique, a method that implies preliminary treatment of chromosomes with trypsin for DNA denaturing and further staining of renatured chromosomes with Giemsa dye [15]. Pairs of homologous chromosomes are arranged according to their number (sex chromosomes on the end) with their short shoulders upwards, ensuring the centromeres are horizontally aligned [16] (Figure 1a). The technique has the potential to elucidate rather large abnormalities exceeding 5 Mb in length and is used today in cases of suspected or existing congenital pathologies, as well as family planning.

More detailed analyses of the genome became possible upon the development of DNA hybridization techniques. At first, tritium-labeled RNA probes were used to search for a specific DNA sequence and the hybrids were detected by autoradiography [17]. Later, the protocol was modified with fluorescent labels replacing the radioactive ones, thus improving safety and decreasing labor consumption [18,19]. Due to its targeted nature, fluorescence in situ hybridization (FISH) (Figure 1b) remains one of the most commonly used techniques to verify variations previously identified by real-time qPCR and multiplex ligation-dependent probe amplification (MLPA). However, its principle has been refined for use in other techniques, such as spectral karyotyping and multicolor FISH, offering new approaches to the visualization and analysis of chromosomes with the use of various combinations of fluorochromes and light filters [20,21].

For whole-genome analysis, comparative genome hybridization was developed in 1992 [22]. In contrast to FISH, where metaphase chromosomes obtained from a patient and DNA probes are complementary to the sequence of interest, the method proposed by Kallioniemi and colleagues isolated and hybridized differentially labeled genomic DNA from the tumor and normal tissue of a patient and utilized chromosome samples from peripheral blood lymphocytes of a healthy volunteer as a reference (Figure 1c). Further refinement of the protocol and development of specialized software for image analysis made the technique more accessible to many laboratories [23]. It has been widely used for the cytogenetic analysis of solid tumors [24]. However, utilization of metaphase chromosomes considerably limits the method resolution (the average size of detected CNVs is 5–10 Mb). Therefore, further research has been aimed at alternative representations of the cytogenetic map.

## 3. Chromosome Microarray Analysis (CMA)

**Figure 2:**
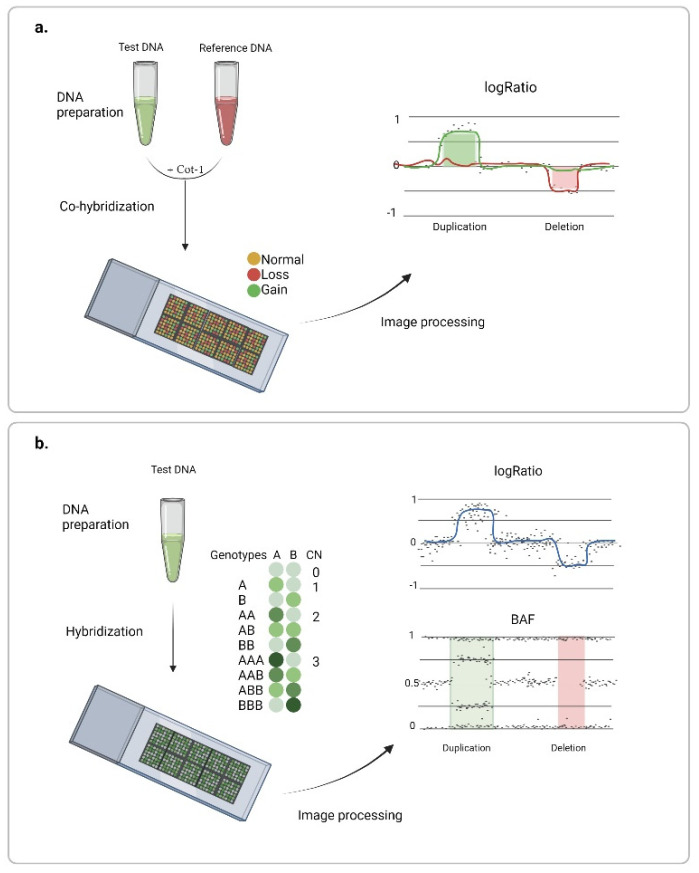
Chromosome microarray analysis: (**a**) array-based comparative genomic hybridization, and (**b**) DNA arrays for genotyping. Created with BioRender.com (accessed on 9 February 2022).

The application of microarrays instead of metaphase chromosomes has provided a solution for large-scale genome studies. Hybridization occurs on multiple DNA probes attached to a solid surface, and physical position on a chip and specific nucleotide sequences of the probes are pre-determined, which allows visualization of relative fluorescence intensities of test and control samples along the genome. For example, in 1997, the matrix-based comparative genomic hybridization method was introduced [25], and soon after, array-based comparative genomic hybridization (aCGH) was introduced [26] (Figure 2a). Resolution of the method depends directly on the probe type, number, and distribution over the genome. At that time, a large set of bacterial artificial chromosome (BAC) clones 80–200 kb long spanning the genome in a fairly complete manner was created in the course of the human genome project [27]. This genomic library has been used for the construction of most CGH arrays capable of identifying variations exceeding 1 Mb [28,29]. In general, any nucleotide sequence can be used as a probe. Later, cDNA- [30,31] and PCR amplicon-based [32] arrays have been successfully used for DNA copy number analysis.

Further improvement of the method occurred upon the deciphering of the whole-genome sequence. This development led to the application of oligonucleotide probes (8–25 bp) that had been previously used for gene expression studies [33,34]. Oligonucleotide probes simplify the process of chip development in regard to both design customization and reproducibility. In addition, these microarrays provide better signal-to-noise ratio and event (CNV) confirmation with several probes due to a more complete genome coverage [35]. Array capacity has also improved, as the mechanical application of DNA onto a chip limited by approximately 50,000 probes has been replaced with oligonucleotide synthesis directly on the glass surface. Commercial technology proposed by Agilent—one of the leading comparative chromosomal analysis technologies today—synthesizes 60-nucleotide probes by an ink-jet technology [36]. Several chip formats have been developed (1 × 1 M, 2 × 400 K, 4 × 180 K, and 8 × 60 K) for whole-genome studies or for the targeted analysis of regions that have been strongly associated with various diseases (Table 1). A higher density of probes is typically used for regions of interest with the distance between adjacent probes as little as several hundreds of nucleotides, and coverage of the rest of the genome provided by evenly spread backbone probes.

Another type of microarray used to search for regions of chromosome imbalance has been developed for genotyping and genome-wide association studies. Here, only fluorescently labeled DNA of the sample is applied to the chip, and copy number analysis is performed based on the rate of target DNA hybridization to allele-specific probes (Figure 2b). Such a technique was first demonstrated in 2004 using the Affymetrix chips [37]. Soon, Illumina introduced an alternative platform, Infinium BeadChip, in which after DNA hybridization occurs on complementary probes, SNPs are evaluated based on the brightness and color of fluorescent nucleotides attached to the probe. The platform could detect deletions and duplications of fragments shorter than 100 kb [38]. However, earlier versions of the chips limited the scope of the search for variation to the regions covering common polymorphisms. Later, the introduction of non-polymorphous probes allowed for more even genome coverage [39]. Today, a wide choice of DNA arrays equipped with 300,000 to several million probes is available on the market (Table 1), including arrays with the possibility of partial or complete customized designs. High genome coverage with short probes provides for higher resolution and more accurate determination of the breakpoints, which makes this type of array a convenient tool when searching for rare and short (starting from 500 bp) variations. Another advantage of the technology is the possibility of identifying mosaicism, loss of heterozygosity (LOH), and uniparent disomies. Soon, the inherent superiority of CMA replaced G-karyotyping as a first-line test in clinical diagnostics of multiple congenital anomalies [40].

Not all chip platforms are equally fit for CNV detection. Although for any type of microarray (CGH, SNP) there is a dependence of the number of identified variations on the total number of probes, the design plays an equally important role [41]. Haraksingh and colleagues demonstrated that a suboptimal number of backbone probes or lack of probes in the intergenic regions results in a disability to identify large, potentially biologically important variations. Further, the comparison of existing platforms shows that additional enrichment with exome variants is not always optimal for CNV detection, as is the case with the Illumina Infinium Omni lineage. However, despite all platform-specific features, the choice of detection algorithm and settings wields the most influence on the results [41,42].

The aim of any algorithm is the analysis of signal intensity along the genome (logR) and elucidating any significant deviations. For this purpose, both simple thresholds, e.g., change of logR by 0.2–0.3 [43,44,45] or by more than 3–4 standard deviations [46,47], and more complex statistical models can be used. Data segmentation methods have found wide applications. For example, Olshen et al. adopted the method of binary segmentation to determine all sites dividing the genome into fragments of the same copy number [48]. In the modified version, two points are determined at each stage, limiting a region so that the t-statistics for the difference in mean probe intensity inside and outside the region are the highest. If the differences are statistically significant, the region is divided, and the procedure is repeated for each of the new three regions, if possible. An alternative method is based on a local genetic search. In this technique, the focus is on the optimization of a certain set of points chosen randomly by means of minimization of the negative logarithmic function of likeness and penalty for the high rate of genome fragmentation [49]. Later on, other methods for change-point detection have been suggested, e.g., ones that are based on the techniques of adaptive weight smoothing [50], fused lasso [51,52], and local search [52,53].

A large class of algorithms utilize hidden Markov models (HMM), allowing for the description of a system with unknown variables based on the observed ones. Changes in the number of copies (loss, gain, or maintenance of genetic material) [54] or absolute number of copies as such [55] are considered hidden states, while their most probable sequence is determined using the dynamic programming after preliminary optimization of the model parameters (the initial probabilities of the states, transition probabilities, and emission probabilities). HMMs are convenient as they work with several variables, as in the case of DNA microarrays. In the latter case, in addition to normalized probe intensities, the ratio of intensities between polymorphous alleles is also under investigation. Additional parameters, such as distance between the probes [56] and the population frequencies of SNPs [57], can be taken into account as well.

Today, multiple ways for microarray data to be processed have been proposed (Table 2). They differ by the number and size of CNVs detected and false-discovery rate [7,42,58,59,60]. As a result, it is recommended to use several algorithms to improve efficiency. Regardless, CMA remains one of the most in-demand methods for CNV detection in both research and clinical diagnostics due to its reliability, flexibility, and relatively low cost. To search for clinically important variants, aCGH or DNA microarrays developed specifically for cytogenetic studies (e.g., Affimetrix Cytoscan HD) and capable of highly reliable identification of variations exceeding 25 kb are preferred.

## 4. Sequencing Data

Methods of CNV detection gained momentum with the rise of high-throughput sequencing (new generation sequencing, or NGS) technology. Based on the analysis of millions of short readings, this was quickly deemed a revolutionary technique due to its high productivity, reproducibility, and accuracy. Data generated from this technique can also be used repeatedly in various types of studies, and this research domain has expanded considerably concerning some genomic variations that were previously difficult to detect. Firstly, the microarray technique had limitations in accessing balanced rearrangements. Secondly, as a consequence of multiple sequencing of random fragments, variation size is not strictly limited as is the case in CMA, where variation less than the distance between two probes cannot be resolved.

**Figure 3:**
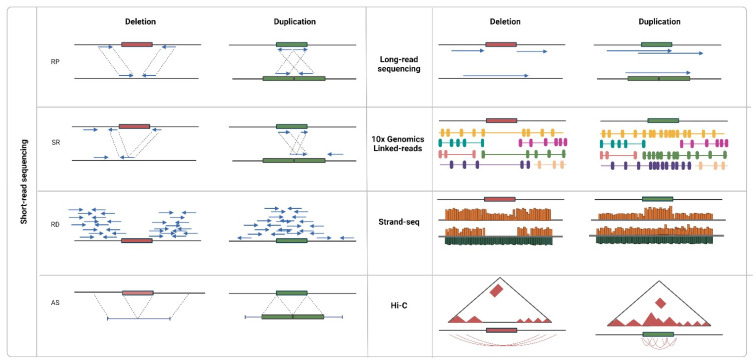
Approaches to CNV detection using sequencing data. Created with BioRender.com (accessed on 9 February 2022).

Detection algorithms are readily being developed in several directions since NGS data can be described by various signatures (Figure 3). One of the first approaches was based on the read pair concept (RP). It implies the presence of aberrations under the condition that distances between the mapped reads on a reference genome reads and/or their orientation is different from the expected ones [65,66]. To search for clusters of such abnormal RPs, two strategies were proposed: in the first case, the distance between the paired reads (insert size) is considered known and constant [67,68], and at least two discordant pairs are required to form a cluster; in the second case, the distribution of insert size for each region over the whole genome is taken into account [69,70]. In the latest studies, concordant read pairs are also taken into account, implying that smaller variations can thus be traced [71].

The split-read (SR) approach also originated from the incoherence property; however, in this case, read is not mapped onto the genome at all or is only partially mapped. The following repeated alignment of the read parts can indicate possible coordinates of the start and end of the variation [72]. The SR approach is suitable for both single and paired reads, but the latter ones impose additional limitations, which accelerate the search. For example, in the Pindel algorithm [73], alignment of the 3′ end of unmapped paired-ends is performed within the double insert size from the 3′ end of mapped paired-ends. The SR approach exists as a rare method to identify deletions 50–100 bp long. Due to its sensitivity to the quality of alignment, it is intended for studies of unique regions of the genome.

In contrast to previous methods indicating only the presence of a variation, the next approach was intended to evaluate the number of copies. The read-depth (RD) approach is based on the assumption that region coverage correlates with the number of its copies. Not limited by either read length or insert size, the approach is suitable for the identification of preferably large variations. The standard detection procedure consists of four stages: mapping of reads and calculation of coverage; normalization; segmentation; and evaluation of the copy number. In the first stage, a genome, or a sliding window, is usually used. The window size can be both chosen voluntarily (for example, 100 nucleotides was considered sufficient for the identification of small variations and accurate search for the breakpoints [74]) or selected based on the data and the desired confidence level of the CNV event [75,76]. Presumably, the number of reads per window is distributed normally; however, in reality, the coverage is shifted in the function of the GC content of the regions [77] and depends on mappability [50]. To take these factors into account, mean normalization methods [78,79,80], as well as various regression models [81,82,83,84], have been proposed. Segmentation and evaluation of copy numbers are performed by most CMA methods, including HMM, mixed Gaussian models, LASSO regression, and CBS.

The RD approach is applicable to the data of whole-exome or targeted sequencing [85]. Although identification of most variations does not seem possible, this type of analysis is convenient in the primary search for patterns specific to a disorder. In addition to the higher coverage of target regions, one should take into account that during genomic library preparation, the efficiency of the enrichment of target regions varies and some regions are over or under-represented. To describe exome data, various models have been proposed, including Gaussian [86], Poissonian [87], beta-binomial [88], and negative binomial [89] distributions (Table 3). In addition, the discrete structure of the data, with rare exceptions, does not allow an analysis of the exact breakpoints of the CNV; however, the analysis can be expanded by using the information on non-target regions that make up 30–40% of sequencing data and providing low genome coverage [78,90]. Another important issue considered in the framing of the problem is the choice of reference samples which are used at the stage of normalization with questions, such as how many reference samples are necessary for efficient detection and are all of them equally useful at being vitally important. The most frequently used strategies include having all available samples sequenced on the same platform with the same chemistry, having all samples in a single sequencing run, or using a set of the most coverage-correlated samples [91].

Recently, the team behind the GATK (Genome Analysis Toolkit), which is known as the most popular tool for analyzing short genomic variations, has also proposed a pipeline to call rare and common germline copy number variants. It uses negative-binomial factor analysis and HMM, and requires at least one-hundred samples to build a model (https://github.com/broadinstitute/gatk/blob/master/docs/CNV/germline-cnv-caller-model.pdf, accessed on 9 February 2022). A somatic mode is also available.

The last approach to detect CNVs using NGS data implies that first, DNA fragments are assembled from overlapping reads de novo, and then the contigs are aligned onto a reference genome (AS). The approach does not require high coverage and is potentially fit for the identification of all types of structural variations, especially new ones. The assembly is performed using graph models (overlapping graphs built by the overlap layout consensus (OLC) method and de Bruijn graph [96] are most often used). Searches for variations can be performed without a reference genome; in such a case, the graph is constructed for several samples and is then analyzed for bifurcations and copy number [97,98].

Despite progress, each of the four described approaches alone is not able to identify the whole range of variations; therefore, the next step was the development of combined methods (Table 4). The most frequently used combinations were RP plus SR or RP plus RD, which are capable of the identification of variations of different lengths and more accurate prediction of breakpoints. Later, algorithms started to take into account the advantages of all three signatures [99,100,101,102,103], which allows a decreased number of false-positive identifications.

Often, CNV detection is narrowed down to classification problems solved by methods of machine learning. Along with various signatures, a number of additional factors, for example, mapping quality (MAPQ) or nucleotide content, are considered [108]. In addition, it is possible to take into account specific features of the formed validation sets, which typically contain intermediate-sized variants. For example, a one-class model trained on a representative set of regions with normal copy numbers searches for regions unlike those in the set, thus covering variations of varying type and size [109]. The latest developments include the DeepSV algorithm based on the analysis of mapped read images [110].

Integration of the data can proceed not only at the level of the signatures, but also at the level of the variants predicted by multiple individual algorithms. Today, the so-called ensemble-based approach is not standardized in any way. Various methods are used for combining and evaluating the variants, including coordinate overlapping, distance between the breakpoints, signature prioritization, agreement of the algorithms, number of confirmations of the event, confidence intervals of the breakpoints, FDR cutoff, and metaheuristics [111,112,113]. Despite the improvement in the accuracy of predictions, all these methods are limited by the characteristics of the input data (short read or insert size), and they do not allow a comprehensive analysis of complex genome regions.

On the contrary, long reads (above 5 kb) produced by third-generation sequencing machines can be used to solve the problem, despite their lack of accuracy. The Pacbio platform performs real-time sequencing of a single molecule through the synthesis of a new strand using a polymerase bound to the well bottom and registration of fluorescence of each newly added nucleotide. In turn, the Oxford nanopore technology is based on the evaluation of change in an electrical current induced by a single-stranded DNA molecule passing through a nanopore. In either case, to detect variations, signatures inside the reads completely covering variations and signatures indicating the presence of a variation based on discrepancies between the reads (orientation, size, or location) are analyzed [114,115] Moreover, calling efficiency depends more on coverage than on the read length or error rate [116].

As an alternative to long reads, the sequencing of bound molecules can be used. For example, using additional barcodes to indicate association to a single DNA molecule, synthetic long reads can be efficient in the detection of large variations. The identification of variants proceeds based on the evaluation of the density of molecule coverage by paired reads, the excessive increase or decrease of which implies a CNV event in a certain region [117,118]. In Strand-seq technology, DNA strands are sequenced independently, and large deletions or duplications are identified based on the evaluation of coverage rate [119]. The size of predicted variations is often limited, which is due to the considerable decrease of coverage resulting from the many purification steps during library preparation. Another method, Hi-C, has been developed to study the 3D structure of chromatin, in particular, to determine the nucleotide sequences that are separately located in the genome but still interact with each other. The matrix of contacts between any two loci can be transformed into the coverage profile with a certain resolution; then, signature-typical analysis methods are applied (normalization, segmentation, and copy number evaluation) [119,120].

In addition to the sequence-based methods mentioned above, optical mapping that aims to determine the physical location of specific sequence motifs or enzymes has great potential for CNV calling. The method first demonstrated in 1993 on the example of construction restriction maps of *Saccharomyces cerevisiae* chromosomes has undergone some modifications [121]. Today’s workflow includes the isolation of high molecular weight DNA, labeling of specific sequences across the entire genome, single-molecule DNA linearization in nanochannels, and imaging using high-resolution fluorescent microscopy. The data obtained can be used for genome assembly improvement [122], haplotype phasing [123], and searches for large structural variations [124]. A CNV event is defined by changes in the density or the distance between restriction sites compared with a reference map obtained from the in silico digestion of the reference sequence. Errors most often occur due to excessive or insufficient stretching of the molecule in the channel, non-specific enzymatic cuts, and incomplete enzyme digestion [125].

## 5. Conclusions

Despite considerable progress, the identification of copy number variations remains a difficult task. Each of the proposed approaches, from cytogenetics to emerging sequencing technologies, capable of the analysis of the so-called dark genome regions has its own limitations. In an attempt to neutralize the latter, over one-hundred methods of analysis have been developed. Particular attention is paid to the development of algorithms aimed at exome and targeted sequencing as optimal tools for applied methods of genome analysis in regard to information load and cost [85], as well as integration of data of any kind, including the use of ensemble models [126,127,128]. Therefore, researchers face a huge solution space from which they usually choose established algorithms, even though they may be less ideal than newer approaches. Algorithms are mainly shaped to detect certain types of variations or length ranges, which should be considered when choosing the approach [129].

Evaluations of the efficiency of existing CNV detection methods and understanding their advantages and limitations are complicated by the lack of a comprehensive validation set. The only available approach for the description of many genomic variations today is the integration of the results of several platforms, as proposed by Zook et al., for simple deletions and insertions [130]. Expansion of the set of platforms used for the analysis and improvement of their accuracy, as well as the development of protocols for integration of the whole bulk of information, are the key problems in this area of research. The design of a variation profile is necessary not only to be used as a reference in the choice of appropriate detection method, but also for basic research focuses, such as the study of the role and function of CNVs, evaluation of their effect on pathogenesis, and many others.

## Figures and Tables

**Table 1:** Modern platforms for chromosomal microarray analysis.

Array Platform	Specification *	Resolution **	Description
Agilent SurePrint G3 Human CGH	1 × 1 M	2.1 kb	enhanced coverage on known genes, promoters, miRNAs, PAR, and telomeric regions
	2 × 400 K	5.3 kb	
	4 × 180 K	13 kb	
	8 × 60 K	41 kb	
Agilent Human Genome CGH	2 × 105	35 kb	
	4 × 44 K	43 kb	
Agilent SurePrint G3 Human Genome CGH + SNP	2 × 400 K	7.2 Kb	
	4 × 180 K	25.3 kb	
Agilent SurePrint G3 Unrestricted CGH ISCA v2	4 × 180 K	25 kb	enhanced coverage on ISCA (International Standards for Cytogenomic Arrays) regions
	8 × 60 K	60 kb	
	4 × 44 K	75 kb	
Agilent SurePrint G3 ISCA v2 CGH + SNP	4 × 180 K	25.3 kb	
Agilent SurePrint G3 Human High-Resolution Discovery	1 × 1 M	2.6 kb	association studies
Agilent SurePrint G3 Human CNV	2 × 400 K	1 kb	
Agilent Human CNV Association	2 × 105 K	232 b	
Agilent SurePrint G3 CGH Postnatal Research	4 × 180 K	2.4 kb	regions identified by Baylor College of Medicine experts
	8 × 60 K	3.7 kb	
Agilent GenetiSure Postnatal Research CGH + SNP	2 × 400 K	9.8 kb	disease-associated regions (The Clinical Genome/ISCA database)
Agilent GenetiSure Pre-Screen	4 × 180 K	31 kb	CNV identification from embryo biopsies and single-cell samples; increased density on chromosomes 13, 18, 20, 21, 22, and X
	8 × 60 K	50 kb	
Agilent GenetiSure Cyto CGH	4 × 180 K	3.5 kb	disease-associated regions linked to developmental delay, intellectual disability, neuropsychiatric disorders, congenital anomalies, or dysmorphic features
	8 × 60 K	7.1 kb	
Agilent GenetiSure Cyto CGH + SNP	4 × 180 K	7.3 kb	
Agilent GenetiSure Cancer Research CGH + SNP	2 × 400 K	9.8 kb	cancer regions of the genome COSMIC (Catalogue of Somatic Mutation in Cancer) CGC (Cancer Genetics Consortium) databases
Illumina HumanCytoSNP	12 × 300 K	6.2 kb	enhanced coverage of ~250 disease regions, including subtelomeric regions, pericentromeric regions, and sex chromosomes
Illumina Infinium CytoSNP-850 K	8 × 850 K	1.8 kb	comprehensive coverage of cytogenetically relevant genes for congenital disorders and cancer research ICCG (International Collaboration for Clinical Genomics) and CCMC (Cancer Cytogenomics Microarray Consortium)
Illumina Infinium Core	24 × 300 K	5.8 kb	genome-wide tag SNPs found across diverse world populations
Illumina Infinium Exome	24 × 300 K	0.21 kb	comprehensive coverage of putative functional exonic variants (including markers representing a range of common conditions, such as type 2 diabetes, cancer, and metabolic, and psychiatric disorders)
Illumina Infinium CoreExome	24 × 600 K	1.82 kb	all of the markers from the Infinium Core-24 BeadChip and the Infinium Exome-24 BeadChip
Illumina Infinium Global Diversity Array	8 × 2 M	0.63 kb	common and low frequency variants in global populations, curated clinical research variants
Illumina Infinium Global Screening Array	24 × 700 K	2.3 kb	multiethnic genome-wide content, curated clinical research variants
Illumina Infinium Omni2.5	8 × 2.4 M	0.65 kb	common and rare SNP content from the 1000 Genomes Project (MAF > 2.5%)
Illumina Infinium Omni2.5Exome	8 × 2.7 M	0.56 kb	combined Infinum Omni2.5 and Infinium Exome-24 markers
Illumina Infinium Omni5	4 × 4.3 M	0.36 kb	comprehensive coverage of the genome including common, intermediate, and rare SNPs
Illumina Infinium Omni5 Exome	4 × 4.6 M	0.33 kb	comprehensive genome-wide backbone combined with putative functional exonic variants
Illumina Infinium OmniExpress	24 × 700 K	2.23 kb	high coverage of common variants for genome-wide association studies
Illumina Infinium OmniExpressExome	8 × 1 M	1.36 kb	tag SNPs and functional exonic content
Illumina Infinium OncoArray	24 × 500 K	5.4 kb	genetic variants associated with five common cancers
Illumina Infinium PsychArray	24 × 700 K	1.74 kb	genetic variants associated with common psychiatric disorders
Affymetrix Genome-Wide Human SNP Array 6.0	1 × 1.8 M	0.68 kb	comprehensive coverage of the genome
Affymetrix CytoScan XON Suite	24 × 6.85 M	0.5 kb	enhanced coverage in 7000 clinically relevant gene, exon-level copy number changes
Affymetrix CytoScan HD	24 × 2.7 M	1.3 kb	enhanced coverage on cytogenetic relevant region

* Samples × No. probes. ** Overall median probe spacing.

**Table 2:** The most widely used algorithms for CNV detection that use microarray data.

Tool	Description	aCGH	SNP-Array		Reference
			Affymetrix	Illumina	
ADM-2	search for intervals in which a Z-score based on the average weighted log ratio exceeds a user-specified threshold	✓			technical documentation (Agilent)
Birdsuite	integration of common CNP genotypes and CNVs discovered using HMM		✓		[61]
ChAS	HMM on the log2 ratios processed through a Bayes wavelet shrinkage estimator		✓		technical documentation (Affymetrix)
cnvPartition	recursive partitioning approach based on preliminary copy number estimates			✓	technical documentation (Illumina)
DNAcopy	circular binary segmentation	✓			[48]
GenoCN	estimation of HMM, parameters from data, germline, and somatic modes		✓	✓	[62]
iPattern	normalization of the total intensities across individuals, Gaussian mixture model fitting		✓	✓	[42]
Nexus	the probe’s log-ratio rank segmentation	✓	✓	✓	[63]
PennCN	HMM, also counted for the population frequency of the B allele		✓	✓	[57]
QuantiSNP	objective Bayes-HMM, fixed rate of heterozygosity for each SNP		✓	✓	[64]

**Table 3:** The most widely used algorithms for whole-exome and targeted data sequencing.

Tool	Description	Data		Mode		Reference
		WES	Targeted	Germline	Somatic	
cn.MOPS	mixture Poisson model and Bayes approach	✓	✓	✓	✓	[92]
CNVkit	in- and off-target regions, rolling median bias correction, CBS	✓		✓	✓	[78]
CODEX	log-linear decomposition-based normalization, Poisson likelihood-based segmentation	✓	✓	✓	✓	[87]
CoNIFER	singular value decomposition-based normalization, ± 1.5 SVD-ZRPKM threshold	✓		✓		[93]
CoNVaDING	ratio scores and Z-scores of the sample of interest compared to the selected control		✓	✓		[94]
DECoN	ExomeDepth modification (the distance between exons is taken into account)		✓	✓		[95]
ExomeDepth	beta-binomial distribution, optimized reference set, HMM	✓	✓	✓		[88]
XHMM	principal component analysis normalization, HMM	✓		✓		[86]

**Table 4:** Combinations of approaches to the analysis of whole-genome sequencing and the most frequently implemented algorithms.

Approach	Tool	Description	Reference
RP	BreakDancer	search for regions that include more anomalous read pairs than expected	[67]
SR	Pindel	pattern growth approach for breakpoint identification	[73]
RD	CNVnator	mean-shift technique, multiple-bandwidth partitioning, and GC correction	[79]
AS	Cortex	bubble-calling in the colored de Bruijn graph	[98]
RP + RD	GenomeSTRiP	connected components algorithm for read pair clustering, Gaussian mixture model for read depth genotyping	[104]
RP + SR	DELLY	graph-based paired-end clustering, breakpoints refinement using split-read alignment	[105]
RP + AS	Hydra	assembly of discordant mate pairs and aligned to the reference genome with MEGABLAST	[106]
RP + SR + AS	Manta	breakend graph construction, independent for each edge variation hypothesis refinement and scoring with diploid model	[103]
RP + SR + RD	Lumpy	probabilistic representation of an SV breakpoint	[102]
Ensemble	MetaSV	merging calls from tools (BreakDancer, CNVnator, BreakSeq, Pindel), breakpoint refinement by aligning the assembled CNV regions	[107]
